# Prediction model for sap flow in cacao trees under different radiation intensities in the western Colombian Amazon

**DOI:** 10.1038/s41598-021-89876-z

**Published:** 2021-05-18

**Authors:** Juan Carlos Suárez, Fernando Casanoves, Marie Ange Ngo Bieng, Luz Marina Melgarejo, Julio A. Di Rienzo, Cristina Armas

**Affiliations:** 1 Facultad de Ingeniería, Programa de Ingeniería Agroecológica, Universidad de la Amazonia, Florencia-Caquetá, Colombia; 2 Facultad de Ciencias Agropecuarias, Maestría Sistemas Sostenibles de Producción, Universidad de la Amazonia, Florencia-Caquetá, Colombia; 3 Centro de Investigaciónes Amazónicas CIMAZ Macagual, Grupo de Investigaciones Agroecosistemas y Conservación en Bosques Amazónicos-GAIA, Florencia, Caqueta ´ Colombia; 4 CATIE - Centro Agronómico Tropical de Investigación y Enseñanza , Turrialba, 30501 Costa Rica; 5 CIRAD, UR Forêts et Sociétés, 34398 Montpellier Cedex 5, France; 6 Departamento de Biología, Laboratorio de Fisiología y Bioquímica Vegetal, Universidad Nacional de Colombia - Sede Bogotá, Bogotá, Colombia; 7 Facultad de Ciencias Agropecuarias, Universidad Nacional de Córdoba, Córdoba, Argentina; 8 Estación Experimental de Zonas Áridas, Spanish National Research Council (CSIC), Carretera de Sacramento s/n, E-04120 La Cañada de San Urbano, Almería, Spain

**Keywords:** Abiotic, Plant physiology

## Abstract

In this study, we measured diurnal patterns of sap flow (*V*_*s*_) in cacao trees growing in three types of agroforestry systems (AFs) that differ in the incident solar radiation they receive. We modeled the relationship of *V*_s_ with several microclimatic characteristics of the AFs using mixed linear models. We characterized microclimatic variables that may have an effect on diurnal patterns of sap flow: air relative humidity, air temperature, photosynthetically active radiation and vapor pressure deficit. Overall, our model predicted the differences between cacao *V*_*s*_ in the three different AFs, with cacao plants with dense Musaceae plantation and high mean diurnal incident radiation (*H*_*PAR*_) displaying the highest differences compared to the other agroforestry arrangements. The model was also able to predict situations such as nocturnal transpiration in *H*_*PAR*_ and inverse nocturnal sap flows indicative of hydraulic redistribution in the other AFs receiving less incident radiation. Overall, the model we present here can be a useful and cost-effective tool for predicting transpiration and water use in cacao trees, as well as for managing cacao agroforestry systems in the Amazon rainforest.

## Introduction

Cacao cultivation is one of the most important agricultural activities in the world; in 2019, 4.8 million tons of beans were produced across 9.9 million hectares^1,2^. Up to 50 million people depend on these cultivations^1^. Cacao is typically grown in tropical climates, mostly under high insolation regimes that impact the cultivation of cacao^3–5^. As it is a tree crop particularly sensitive to drought^6,7^, shading improves the microclimatic conditions where cacao trees grow, consequently improving the plants’ water status^8^ that has an overall positive impact on crop production^9,10^. For these reasons, 70% of the area covered by cacao plantations throughout Latin America is managed through agroforestry arrangements where cacao crops are planted under the shade of other trees, or of annual and evergreen crops^11^.

Modelling transpiration fluxes of target tree crops is key to analyzing the water balance of any agroforestry system (AF). Transpiration is generally analyzed using the Penman–Monteith adjusted equation that is based on, among other inputs, field measurements of sap flow^12–14^. In such models, transpiration is calculated using direct measures of the diurnal accumulated sap flow density, a modeling approach that has been applied for many major crops including cacao^15–18^, coffee^19–22^, apple^23,24^, papaya^25,26^, in vineyards^27,28^ and in other tropical tree crop species^29^.

Diurnal sap flow measurements are costly, both in terms of equipment and data post-processing^30,31^. Thus, some efforts have been made to model diurnal sap flow, such as the Jarvis-type model^32^, which predicts sap flow by using non-linear models that depend on a single environmental factor (e.g., photosynthetically active radiation or vapor pressure deficit^16,18^) or a group of environmental variables^33^. All such models consider that vapor pressure deficit (*VPD*) and photosynthetically active radiation (*PAR*) are the microclimatic variables that have the largest impact on sap flow; however, this assumption may be an over-simplification^16,17,34^. In a cacao AF, the shade of companion species impacts many other microclimatic variables such as incident radiation, diurnal amplitude and mean temperature, and relative air humidity. These microclimatic variables may also impact the diurnal production and transpiration of the crop, having effects on the crop that could be independent of the ones caused by *VPD* or *PAR* alone. In such cases, accurate sap flow predictions require more complex models that take into account the simultaneous effects of these key microclimatic variables, which are affected by the modification of the AF structure. Once these models are developed, they can be used to determine diurnal patterns of tree sap flow in each type of AF; posterior calculations of sap flow would then only require the measurement of these microclimatic variables. These models would ultimately be more affordable and practical tools to calculate transpiration and water use of single cacao trees^15–18,33^ as well as of the entire cacao plantation grown in different AFs. Blooming in cacao trees occurs during the relative dry season, and flower and fruit set productions would depend on plant water status and C assimilation rates^35^. Diurnal sap flow rates are a proxy of diurnal tree transpiration rates, gas exchange and C fixation^36,37^ that, during the onset of the reproductive period, can be assigned to flower and fruit production. Thus, those AFs displaying adequate cacao sap flow dynamics during cacao reproductive period would increase the production of flowers^38^ and fruit set^39^.

The overall aim of the study is to propose a cost-effective model to predict sap flow in cacao trees based on multiple microclimatic variables relevant to cacao AFs in the continental Amazon region during the relative dry period when cacao trees flower and fructificate. The climate and overall environmental conditions of cacao plantations are similar in this region, and all plantations use similar companion species to arrange the agroforestry structure. More specifically, in this study we measured diurnal cacao sap flow (*V*_s_) and modelled it based on different microclimatic variables (air relative humidity, air temperature, photosynthetically active radiation, and *VPD*). We then analyzed the diurnal patterns of sap flow movement and day–night directions of *V*_s_ in three cacao AFs in the Continental Amazon region. These AFs differed in the density and type of companion species, generating different diurnal incident radiation intensities among AFs. After the validation of the *Vs* model, we discussed the interest of this modeling approach, specifically in relation to its usefulness for calculating diurnal water use of cacao plantations under different AF managements, as well as its potential effects on cacao production.

## Materials and methods

### Study site and AF characteristics

**Figure 1:**
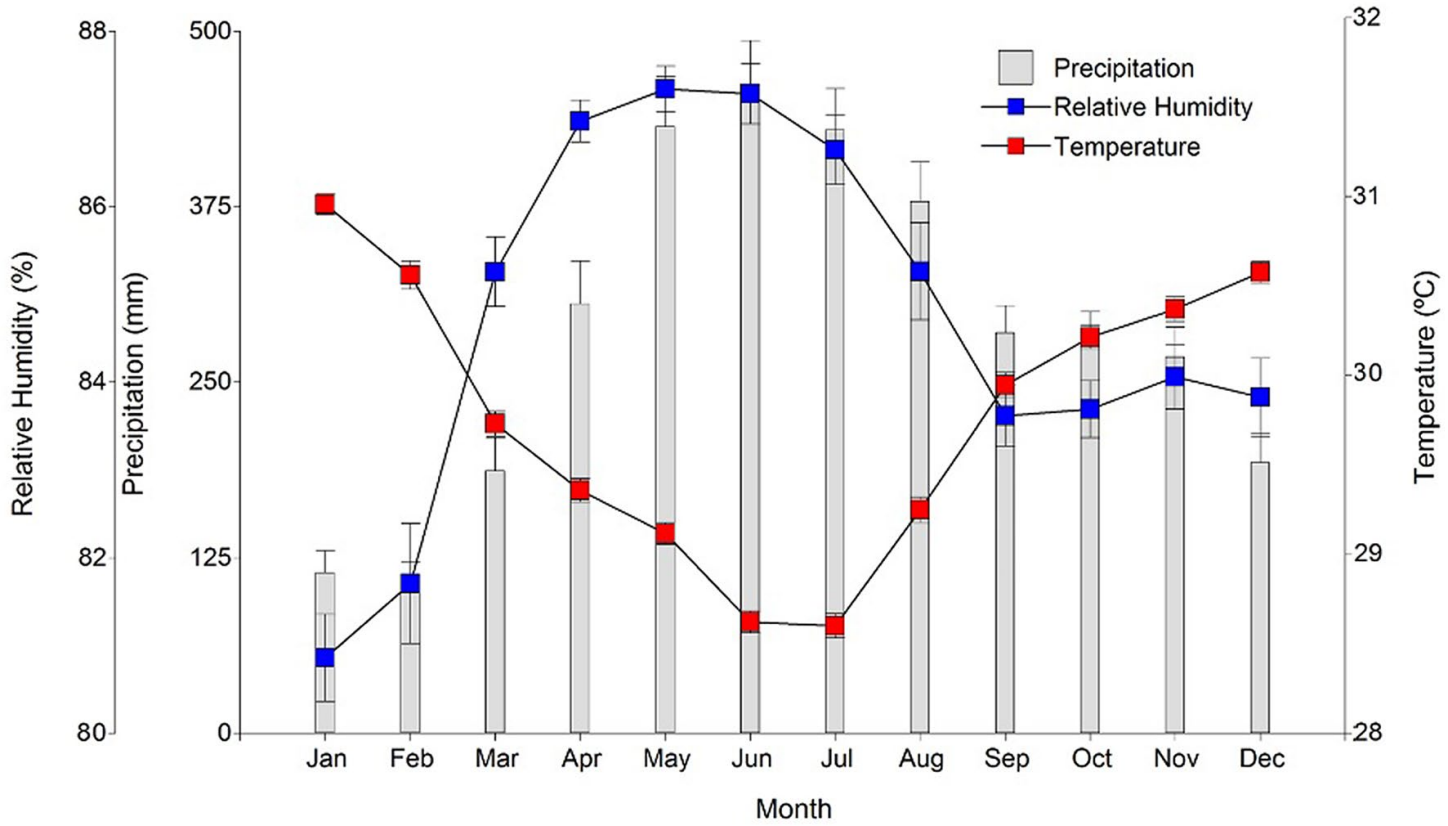
Climate diagram of the Continental Amazon region, including monthly precipitation, mean monthly temperature, and air relative humidity (values calculated based on the last 30 years of climatic data). The driest month is January (high air temperature, low precipitation, and low air relative humidity).

Sap flow measurements were taken from cacao trees in three agroforestry systems at the Centro de Investigaciones Amazónicas (Amazonian Investigations Center) CIMAZ Macagual—Universidad de la Amazonia (137′ N and 7536′ W at 360 m a.s.l.), Colombia. The three AFs differed in structure (number and type of companion species) and incident radiation (see below). The climate is warm–humid, characteristic of Amazonian tropical rainforest ecosystems, with a mean annual precipitation of 3443 mm, 1200 sunshine hours per year, a temperature between 28.5 and 31 °C and a relative humidity between 81 and 88% (Fig. 1). Sap flow was measured over the course of two weeks during the period of minimum rainfall in the region (November to February). We selected this period for measurements because cacao plant production, transpiration, flowering and fruit-set differed the most across AFs during this relatively dry season, and adequate cacao response to potential drought spells is of great economic importance. Anyhow, as major abiotic characteristics (climate, soil and topography) were equal in all AFs, and the period studied was a relatively mild dry period, we did not expect major differences in soil water contents across AFs, and expect that major differences in cacao physiological responses across AFs would be caused by the different microclimatic conditions and incident radiation among AFs. During the rest of the year, the climate is particularly humid and cloudy, and a preliminary analysis of cacao tree physiological responses across AFs did not render significant results.

**Figure 2:**
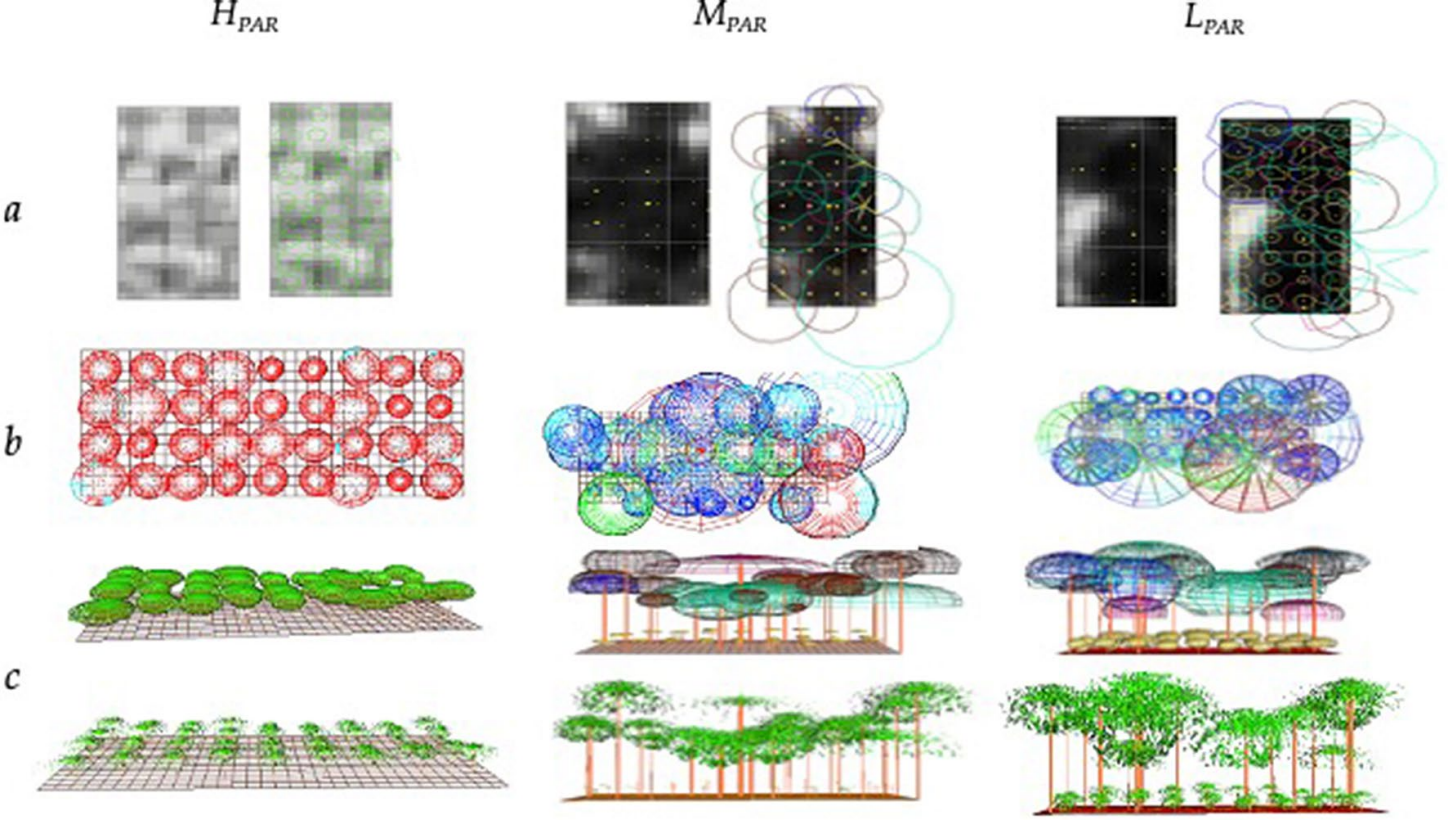
Digital models of the horizontal and vertical structure of each type of agroforestry system (AF) studied. The grayscale in (**a**) corresponds to the level of shadow generated by the shade canopy (darker corresponds to less incident radiation); upper or horizontal (**b**) and lateral or vertical (**c**) profiles of the agroforestry systems with each of the companion species defined with different colors. Digital models were built with SExI-FS^40^ and Shademotion^41^ software’s. The agroforestry arrangements are: cacao AF with dense Musaceae plantation and high mean diurnal incident radiation (H_PAR_); cacao AF with low density of clustered vegetation and medium mean diurnal incident radiation (M_PAR_); and cacao AF with high density diversified multistrata vegetation and low mean diurnal incident radiation (L_PAR_). Other characteristics of the AFs are described in methods and Table 1.

The three AF plots analyzed in this study were 25 × 50 m in size. The cacao plants were all clones (and thus of the same age), planted in the AFs in October 2012 in a regular pattern with a distance of 3 m between plants, irrespective of the AF. Although the cacao plantation was identical in the three plots, each AF differentiated itself based on the type and density of the companion species (henceforth called vegetation), which were planted in 2008 in rows with a north–south orientation and created an upper canopy with varying levels of shade. Two of the AFs included companion timber species (*Cariniana pyriformis*, *Calycophyllum spruceanum*), and a third AF included Musaceae species (plantain; *Musa paradisiaca*). The three cacao AFs compared in this study were (Fig. 2; Table 1):

i. *Cacao AFs with dense Musaceae plantation and high mean diurnal incident radiation* (*H*_*PAR*_) with temporary shade generated by plantain plants, planted at a density of 527 plants ha^−1^ (i.e., one plantain plant per 6 × 3 m area). The diurnal mean *PAR* transmitted to cacao was 700 µmol m^−2^ s^−1^ (with mean midday values of 1300 µmol m^−2^ s^−1^);
ii. *Cacao AFs with a low density of clustered vegetation and medium mean diurnal incident radiation* (*M*_*PAR*_) with average shade that is generated by trees with thin crowns (*Cariniana pyriformis*, *Calycophyllum spruceanum*), planted at a density of 35 trees ha^−1^ (i.e., one tree per 12 × 25 m area). The diurnal mean *PAR* transmitted to cacao was 400 µmol m^−2^ s^−1^ (with mean midday values of 900 µmol m^−2^ s^−1^);
iii. *Cacao AFs with high density multistrata vegetation and low mean diurnal incident radiation* (*L*_*PAR*_) with intense shade, generated by the same trees as M_PAR_ (*Cariniana pyriformis*, *Calycophyllum spruceanum*) but planted at a density of 55 trees ha^−1^ (i.e., one tree per 12 × 15 m area). There is a diurnal mean *PAR* transmitted to cacao was 300 µmol m^−2^ s^−1^ (with mean midday values of 500 µmol m^−2^ s^−1^).

**Table 1:** Different measurements done in cacao trees (*Theobroma cacao*) and accompanying species in each of the agroforestry systems (AFs) analyzed in this study.

	Agroforestry system		
	H_PAR_	M_PAR_	L_PAR_
**Cacao trees**			
*Theobroma cacao*			
Main stem diameter (cm)	7.32 ± 0.52	7.22 ± 0.45	7.14 ± 0.36
Height (m)	2.29 ± 0.03	2.15 ± 0.02	1.96 ± 0.02
Crown diameter (m^2^)	7.42 ± 0.37	7.19 ± 0.52	7.11 ± 0.17
**Shade canopy accompanying species**			
*Cariniana pyriformis*			
DBH	–	0.31 ± 0.02	0.35 ± 0.04
Height (m)	–	9.75 ± 0.25	10.5 ± 0.82
Crown diameter (m^2^)	–	8.1 ± 0.4	13.3 ± 1.2
*Calycophyllum spruceanum*			
DBH	–	0.15 ± 0.01	0.18 ± 0.04
Height (m)	–	11.5 ± 0.67	11.38 ± 0.75
Crown diameter (m^2^)	–	5.6 ± 0.57	6.6 ± 0.58

These AFs mainly differ in the density and species shading the cacao crop and thus in the incident radiation the cacao crop receive and the associated microclimatic changes. Tree measurements are, main stem diameter (cacao; measured at 20 cm above ground) or diameter at breast height (DBH, rest of the trees), tree height, and crown diameter. Main characteristics of AFs and their legend are described in the methods section and Fig. 2.

### Monitoring sap flow in the sampled cacao trees

Sap flow was quantified using the SFM1 sap flow meter (ICT International Pty Ltd., Armidale, Australia) installed in the main trunk of four randomly chosen cacao plants in each AF at a height of 20 cm from the ground. Because the plants are clones of the same age, we assumed that differences recorded at the physiological level between systems are due to the effect of the AFs on the microclimatic variables under the canopy, as well as to the effects of these variables on the physiological behavior of cacao, absent confounding factors. Continuous sap flow measurements were made during the end of the dry season, between January 13 and 27 of 2015. These sensors use the Heat Ratio Method (HRM), which was developed and presented by Burgess et al*.*^42^. Each sensor consists of two thermocouples spaced 15 mm apart and inserted in the main stem at 5 mm and 20 mm depth behind the cambium. In order to calibrate using a reference value of the sap flow velocity (zero), we used the procedure suggested by Burgess et al.^42^ which consists of cutting the stem below the measuring needles and recorded sap velocity (*V*_h_) afterwards. All corrections for wounds and misalignment of the probes were made according to Burgess et al*.*^42^. The velocity of the heat pulse (*V*_h_) can be calculated as a function of different parameters as:

1 $${V}_{h}=\frac{k}{x} ln\left({T}_{1}/{T}_{2}\right)3600$$

where: *V*_h_ is the heat pulse velocity (m s^−1^), *k* is thermal diffusivity of green (fresh) wood (with a default value of 0.0025 cm^2^ s^−1^^43^), *x* is the distance between the heater and each of the thermocouples needles (cm; 0.6 cm for this particular sensors), and *T*_*1*_ and *T*_*2*_ are the temperatures (°C) in the position of the needles, located at equidistant points downstream and upstream from the heater. Heat pulse velocity (*V*_h_) was converted into sap flux density (*V*_s_ sap flow rate L h^−1^) using the equation proposed by Burgess et al.^42^, as:

2 $${V}_{s}=\frac{{}_{b}}{{}_{s}} \left({m}_{c}+\frac{{C}_{w}}{{C}_{s}}\right)*{V}_{h}*\mathrm{ S}$$

where ${}_{b}$ is the basic density of the dry sapwood (i.e. dry sapwood weight divided by its green volume), ${}_{s}$ is the sap density (assumed to be the density of water), ${m}_{c}$ is the water content of the fresh sapwood, ${C}_{w}$ is the specific heat capacity of the dry wood matrix (1200 J kg^−1^ K^−1^ at 20 °C^44^), ${C}_{s}$ is the specific heat capacity of the sap (assumed to be that of water, 4186 J kg^−1^ K^−1^ at 20 °C^44^), and S is the cross-sectional area of the conducting sapwood.

Raw heat pulse velocity data collected by the SFM1 Sap Flow Meters were imported into the Sap Flow Tool software (ICT International, Armidale, NSW, Australia) that calculated sap flow, diurnal sap flow and cumulative sap flow rates (L h^−1^) using the above mentioned functions.

### Monitoring microclimatic variables in the AFs under study

A WatchDog 2900ET weather station (Spectrum Technologies, Inc., USA) was placed under the canopy at a height of three meters, and moved within the plot every day to monitor the microclimatic parameters: air relative humidity (*RH*_*a*_), air temperature (*T*_*a*_), and photosynthetically active radiation (*PAR*) at a frequency of once per minute. The vapor pressure deficit (*VPD*) was calculated based on air temperature and air relative humidity, which were recorded minute by minute using the methodology proposed by Allen et al*.*^45^ which records the maximum and minimum temperature and humidity values during a given period.

### Modelling sap flow under different radiation intensities

#### The model

We first compared the microclimatic variables across AFs using linear mixed models (LMM) where *RH*_*a*_, *T*_*a*_, *PAR*, and *VPD* were the responses variables, AFs was the treatment factor, and day and plant effects were considered random factors. Mean values were compared using Fisher’s LSD post-hoc test (α = 0.01). The assumptions of normality and homogeneity of variance were evaluated using an exploratory analysis of residuals. Sap flow models were then adjusted using the totality of the data recorded during the monitoring period (n = 3816 measurements of sap flow in the four cacao plants of each AF). To predict *V*_*s*_ (sap flow, dependent variable) based on microclimatic variables (predictor variables), new LMM were adjusted. Type of AF was included in the model as a dummy variable, and *RH*_*a*_, *T*_*a*_, *PAR*, and *VPD* as predictors. The model for each AFs was:

3 $$V_{s} = \beta_{0} + \beta_{1} RH_{a} + \beta_{2} T_{a} + \beta_{3} PAR + \beta_{4} VPD$$

Bayesian Information Criterion (BIC) and Akaike Information Criterion (AIC) were used as criteria for the selection of the best model. Partial residuals were studied to verify the type of relationship (i.e., linear or quadratic) to be included between each microclimatic variable and the response variable. The adjustments to the LMM were made using the function *lme*^46^ in the package *nlme* of R-Project version 3.6.0^47^ and using the implemented interface of the R platform in InfoStat^48^.

#### Validation

A simple linear regression analysis was conducted between predicted values, generated using the best (AIC/BIC) model for sap flow, and a validation dataset (n = 1740). The R^2^ value was considered a measure of the predictive value of the model. We used the methodology of Vezy et al*.*^49^ to evaluate the goodness of fit of the model. In order to evaluate the mean precision of the model with the same units as the variable of interest, we calculated RMSE and bias. The modeling efficiency (EF), also known as Nash–Sutcliffe efficiency (NSE), was also used to describe how well the graph of observed versus simulated data fits in the identity function. A standard regression was used to describe the relative relationship between the observations and the simulation (slope), which allows us to identify any lag or deviation between the simulated and observed values. For this purpose, scatter plots were created showing the fit line and the best fit line (i.e., y = x). The goodness of fit of the model was evaluated using the function *ggof*^50^ in the package *HydroGOF* of R-Project version 3.6.0^47^ and using the implemented interface of the R platform in InfoStat^48^.

## Results

### Sap flow variability under different radiation intensities

**Figure 3:**
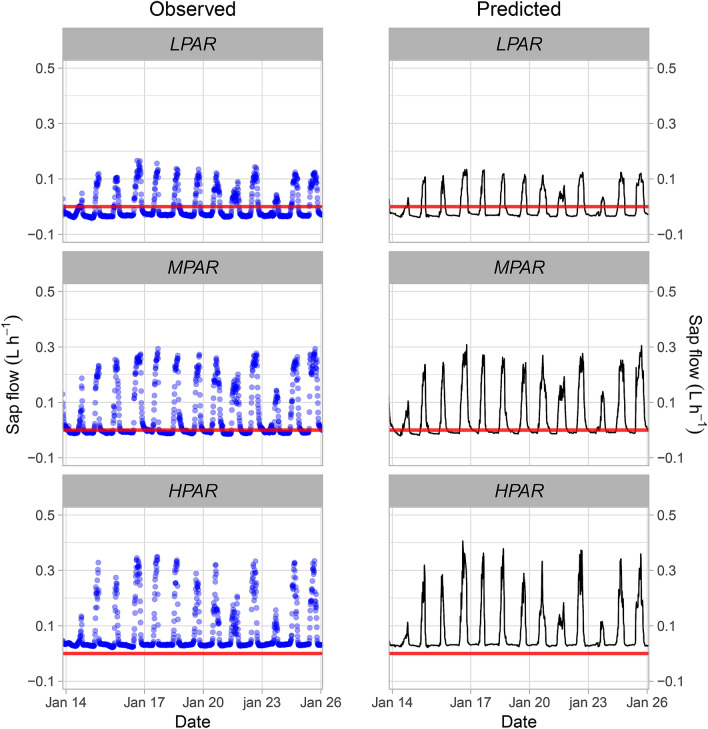
Diurnal pattern of sap flow of cacao plants under different agroforestry arrangements: (**a**) observed, and (**b**) predicted sap flow based on the LMM model. The agroforestry arrangements are described in the methods section and Fig. 2.

In the *H*_*PAR*_ agroforestry system, the cacao plant exhibited increasing sap flows throughout the entire monitoring period, even at night (Fig. 3a). The maximum sap flow average values were 0.27 ± 0.03 L h^−1^ during daylight hours and 0.0300 ± 0.0023 L h^−1^ during the night. The AFs *M*_*PAR*_ and *L*_*PAR*_, however, exhibited negative sap flows at night, with average values of − 0.0047 ± 0.0085 L h^−1^ and − 0.0314 ± 0.004 L h^−1^, respectively (Fig. 3a). For *M*_*PAR*_ and *L*_*PAR*_, nocturnal averages were significantly different from zero (t-test; *p* < 0.01), which may indicate a differential nocturnal hydraulic redistribution across these agroforestry arrangements. When comparing sap flow under different intensities of sun radiation, sap flow was higher in *H*_*PAR*_ compared to the treatments, with maximum values recorded at noon. Predicted values obtained from our LMM model closely resembled the observed data (Fig. 3a,b; see below).

### Monitoring microclimatic variables in the AFs under study

The air relative humidity was, on average, 90% with a variation range between 49 and 100% (Table 2). The mean air temperature was 21.4 °C at night and 28.1 °C during the day. The mean *PAR* value during the day was 708.9 µmol m^−2^ s^−1^, with a maximum value of 2310 µmol m^−2^ s^−1^. Mean *VPD* was 0.28 kPa, with a maximum value of 1.86 kPa. As illustrated in Table 1, the greatest differences between AF arrangements in all microclimatic variables occurred at 13:00 h (solar time) and were significant in every case (*p* < 0.01); differences between arrangements in all microclimatic variables were also recorded at 10:00 and 16:00 h (*p* < 0.01). In the case of the *L*_*PAR*_ arrangement, the average temperature at 13:00 h was 26.6 °C—the minimum value recorded in any of the three intensities. On average, temperatures in *M*_*PAR*_ and *H*_*PAR*_ were 1.81 °C and 3.12 °C higher, respectively (Table 1). There were also differences in all microclimatic variables during the night, and these differences were significant in all cases (*p* < 0.01); specifically, at 04:00 h, there were significant differences for *T*_*a*_ and *VPD* between agroforestry systems.

**Table 2:** Mean values ± SE of the microclimatic variables during the monitoring period under different radiation intensities and at different hours of the day (official hours UTC/GMT—5:00 h): air relative humidity (*RH*_*a*_, %), air temperature (*T*_*a*_, °C), photosynthetically active radiation (*PAR*, µmol m^−2^ s^−1^), vapor pressure deficit (*VPD*, kPa).

Hour	Arrangement	*RH*_*a*_				*T*_*a*_				*PAR*				*VPD*			
		Mean		S.E		Mean		S.E		Mean		S.E		Mean		S.E	
Day																	
10:00	*L*_*PAR*_	88.32	±	1.91	a	23.98	±	0.43	c	354.51	±	38.66	b	0.30	±	0.05	b
	*M*_*PAR*_	81.69	±	2.66	b	25.88	±	0.49	b	446.25	±	51.58	b	0.53	±	0.08	a
	*H*_*PAR*_	80.26	±	3.18	b	26.87	±	0.67	a	1426.98	±	216.07	a	0.56	±	0.10	a
13:00	*L*_*PAR*_	76.56	±	2.69	a	26.63	±	0.64	c	467.67	±	45.80	b	0.69	±	0.09	c
	*M*_*PAR*_	69.16	±	3.10	b	28.44	±	0.67	b	559.43	±	51.88	b	1.00	±	0.12	b
	*H*_*PAR*_	67.94	±	3.45	b	29.75	±	0.80	a	2278.98	±	275.40	a	1.12	±	0.15	a
16:00	*L*_*PAR*_	80.11	±	3.49	a	26.25	±	0.86	c	216.88	±	37.74	b	0.61	±	0.13	b
	*M*_*PAR*_	76.51	±	4.02	c	27.03	±	0.81	b	238.44	±	38.69	b	0.75	±	0.15	a
	*H*_*PAR*_	78.24	±	3.93	b	27.47	±	0.89	a	778.36	±	148.99	a	0.71	±	0.14	a
Night																	
20:00	*L*_*PAR*_	97.86	±	0.19	a	23.04	±	0.17	a					0.05	±	0.01	b
	*M*_*PAR*_	95.57	±	0.37	b	23.27	±	0.12	a					0.11	±	0.01	a
	*H*_*PAR*_	95.06	±	0.38	b	23.65	±	0.12	b					0.12	±	0.01	a
00:00	*L*_*PAR*_	99.47	±	0.08	a	21.99	±	0.16	a					0.01	±	0.01	b
	*M*_*PAR*_	98.41	±	0.22	b	22.43	±	0.12	b					0.04	±	0.01	a
	*H*_*PAR*_	98.03	±	0.18	b	22.79	±	0.12	b					0.05	±	0.01	a
04:00	*L*_*PAR*_	99.84	±	0.05	a	21.48	±	0.13	a					0.00	±	0.00	a
	*M*_*PAR*_	99.35	±	0.09	b	22.21	±	0.11	a					0.01	±	0.01	b
	*H*_*PAR*_	99.03	±	0.09	c	22.44	±	0.10	b					0.05	±	0.01	c

The agroforestry arrangements are described in the methods section and Fig. 2. Values in a column that have different letters within the same time slot indicate significant differences between AFs (post-hoc LSD tests, *p* < 0.05).

**Figure 4:**
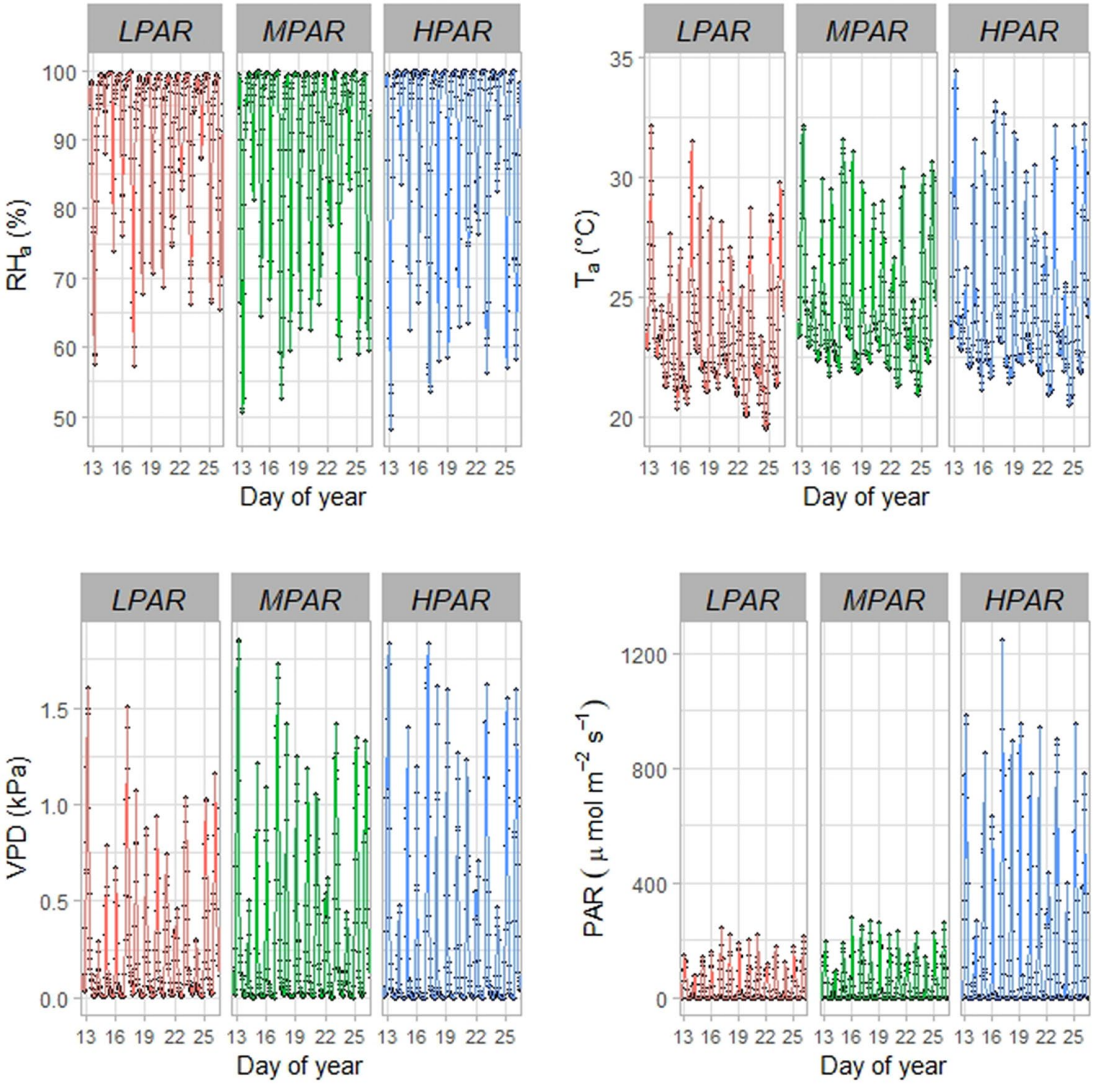
Diurnal patterns of air relative humidity (*RH*_*a*_); air temperature (*T*_*a*_); vapor pressure deficit (*VPD*); and photosynthetically active radiation (*PAR*) under different agroforestry arrangements during the period of study. Types of AFs and legend are described in the methods section and in Fig. 2*.*

For the remaining variables, differences between agroforestry arrangements followed similar patterns. The lowest values of transmitted *PAR* and *VPD* were recorded in *L*_*PAR*_, where there is a denser companion tree canopy; the highest values were recorded in *H*_*PAR*_, and intermediate values in *M*_*PAR*_. Relative humidity followed the opposite pattern (maximum in *L*_*PAR*_, minimum in *H*_*PAR*_). We noticed that the *PAR* values at 13:00 h in the *H*_*PAR*_ arrangement (2279 µmol m^−2^ s^−1^) had an mean value higher than those values obtained in the other two arrangements (Table 2; Fig. 4). When relating the sap flow values measured in the different plots to the different microclimatic variables, we found a negative correlation between *V*_*s*_ and *RH*_*a*_, and a positive correlation between *V*_*s*_ on the one hand, and *PAR*, *T*_*a*_ and *VPD* on the other (all had correlation coefficients greater than 0.73 in general terms, and greater than 0.78 within each plot, ranked r(*V*_*s*_*,PAR)* > r(*V*_*s*_*,VPD)* > r(*V*_*s*_*,T*_*a*_)) (Table 3).

**Table 3:** Pearson correlation coefficients between *V*_*s*_ in cacao plants and microclimatic variables during the monitoring period under different radiation intensities.

Factor	*RH*_*a*_	*T*_*a*_	*PAR*	*VPD*
General	− 0.78	0.73	0.75	0.77
*L*_*PAR*_	− 0.87	0.78	0.91	0.84
*M*_*PAR*_	− 0.93	0.90	0.90	0.92
*H*_*PAR*_	− 0.92	0.88	0.93	0.92

Types of AFs are described in the methods section and in Fig. 2*.* The microclimatic variables are: air relative humidity (*RH*_*a*_); air temperature (*T*_*a*_); photosynthetically active radiation (*PAR*); and vapor pressure deficit (*VPD*). All correlation coefficients were highly significant (*p* < 0.0001).

### Modelling sap flow (V_s_) under different radiation intensities

**Figure 5:**
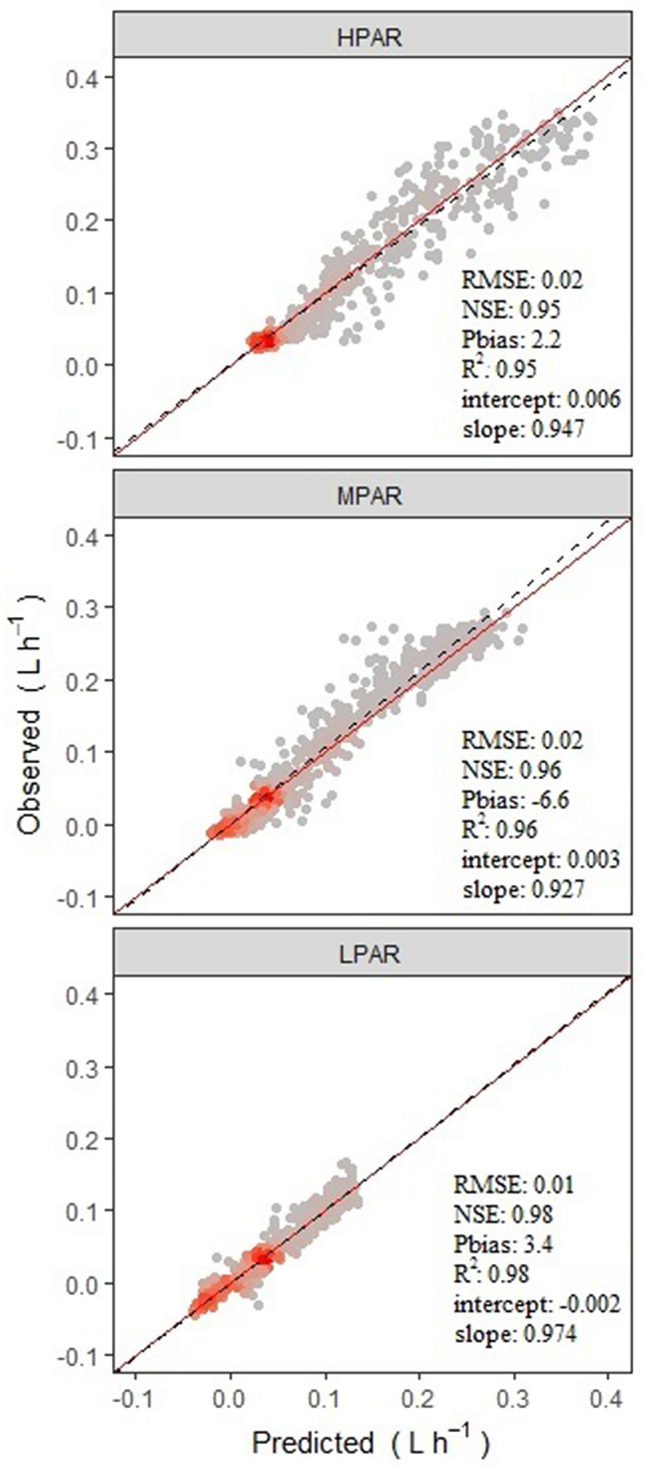
Regression line between observed *V*_*s*_ (y-axis) versus predicted *V*_*s*_ (x-axis) obtained from the LMM model for each agroforestry system (*p* < 0.0001 in all cases) are shown as dotted lines (red line represents y = x). The Root Mean Square Error (RMSE), Nash–Sutcliffe efficiency (NSE), Percent Bias (Pbias), and coefficient of determination (R^2^) are given. Colors represent a gradient of density of the points from grey (the lowest) to red (the highest). Types of AFs and their legend are described in the methods section and in Fig. 2.

The general model which estimated the relationship between observed versus predicted *V*_*s*_ for all three AFs, showed that the estimate was very close to the y = x line (Fig. 5a–c). The model allowed us to explain the diurnal behavior of sap flow, which was affected by the microclimatic variables within each agroforestry arrangement that, in turn, impacted the incident radiation intensity that reached the plot. Specifically, the model predicted the water use of the cacao crop as a function of time, replicating phenomena such as hydraulic redistribution (HR). Indeed, *V*_*s*_ was predicted well by the statistical model (R^2^ = 0.98) that included the microclimatic regressors and exhibited significant differences between the three different AFs (Tables 4, 5). Significant differences were found between the slopes of all AFs for each microclimatic variable (*RH*_*a*_, *T*_*a*_, *PAR*, *VPD*), with values of *p* < 0.01 except for *T*_*a*_ between *H*_*PAR*_ and *M*_*PAR*_ (*p* > 0.05). Differences in intercepts were also observed depending on the AFs (*p* < 0.01). When analyzing the behavior of *V*_*s*_ and the process of HR, we found that the microclimatic variables that determine this phenomenon were the *RH*_a_ and *VPD*. This was due to the negative slopes in the model for both *RH*_a_ and *VPD* in the radiation intensities *M*_*PAR*_ and *L*_*PAR*_, which were statistically different from *H*_*PAR*_.

**Table 4:** Coefficients of the best model (according with AIC and BIC criterion) predicting sap flow (*V*_*s*_) as a function of a set of microclimatic variables across the different radiation intensities for cacao plantations in the Colombian Amazon.

	Value		Std. Error	t-value	*p* value
*H*_*PAR*_	0.14904	±	0.02786	534.879	< 0.0001
*M*_*PAR*_	0.37090	±	0.03559	1.042.063	< 0.0001
*L*_*PAR*_	− 0.04641	±	0.02349	− 197.595	0.0482
M_PAR_ vs L_PAR_	0.41732	±	0.02908	20.592.275	< 0.0001
*RH*_*a*_* H*_*PAR*_	− 0.00059	±	0.00022	− 272.954	0.0064
*M*_*PAR*_	− 0.00409	±	0.00033	− 1.249.385	< 0.0001
*L*_*PAR*_	− 0.00057	±	0.00021	− 265.782	0.0079
*M*_*PAR*_* vs L*_*PAR*_	− 0.00352	±	0.00027	17.127.910	< 0.0001
*T*_*a*_* H*_*PAR*_	− 0.00258	±	0.00046	− 558.407	< 0.0001
*M*_*PAR*_	− 0.00043	±	0.00036	− 119.058	0.2339
*L*_*PAR*_	0.00178	±	0.00027	658.568	< 0.0001
*M*_*PAR*_* vs L*_*PAR*_	− 0.00220	±	0.00030	5.416.543	< 0.0001
*PAR H*_*PAR*_	0.00007	±	0.00001	1.460.565	< 0.0001
*M*_*PAR*_	0.00050	±	0.00001	3.558.999	< 0.0001
*L*_*PAR*_	0.00001	±	0.00001	0.94846	0.3430
*M*_*PAR*_* vs L*_*PAR*_	0.00049	±	0.00001	135.358.210	< 0.0001
*VPD H*_*PAR*_	0.12195	±	0.00697	1.749.240	< 0.0001
*M*_*PAR*_	− 0.07844	±	0.01113	− 704.621	< 0.0001
*L*_*PAR*_	− 0.06120	±	0.00721	− 849.277	< 0.0001
*M*_*PAR*_* vs L*_*PAR*_	− 0.01724	±	0.00913	356.462	0.0591

The types of AFs and legend are described in the methods section and Fig. 2*.* The microclimatic variables are: air relative humidity (*RH*_*a*_, %); air temperature (*T*_*a*_, °C); photosynthetically active radiation (*PAR*, µmol m^−2^ s^−1^); and vapor pressure deficit (*VPD*, kPa). The types of AF were included as dummy variables in the regression model to control for potentially effects of different agroforestry arrangements and incident radiation intensities on microclimatic variables and sap flow across AFs. The first three coefficients (Value) shown in the table are: the value for *H*_*PAR*_ corresponds to β_0_ (see Eq. 3) in *H*_*PAR*_, the value for *M*_*PAR*_ corresponds to the difference between *H*_*PAR*_-β_0_ and *M*_*PAR*_-β_0_*,* the value for *L*_*PAR*_ corresponds to the difference between *H*_*PAR*_-β_0_ and *L*_*PAR*_-β_0_. The following groups of coefficients (row blocks separated by a line) correspond to the coefficients for each microclimatic variable and is interpreted in the same way as described above but applied to the partial slope for each microclimatic variable. In all cases, the *M*_*PAR*_ vs *L*_*PAR*_ rows show the difference between their estimated coefficients.

**Table 5:** Coefficients of the model (see Eq. 3) for the determination of *V*_*s*_ of cacao trees using microclimatic variables under different radiation intensities as predictors.

Coefficients	*H*_*PAR*_	*M*_*PAR*_	*L*_*PAR*_
β_0_	0.14904	0.37090	− 0.04641
*RH*_*a*_	− 0.00059	− 0.00468	− 0.00116
*T*_*a*_	− 0.00258	− 0.00301	− 0.0008
*PAR*	0.00007	0.00057	0.00008
*VPD*	0.12195	0.04351	0.06075

Types of AF are described in the methods section and in Fig. 2*.* Microclimatic variables are: air relative humidity (*RH*_*a*_; %); air temperature (*T*_*a*_; °C); photosynthetically active radiation (*PAR*; µmol m^−2^ s^−1^); and vapor pressure deficit (*VPD*; kPa). Coefficient values were calculated using results in Table 4. β_0_ is the intercept.

An analysis of the predicted values versus the observed values produced a coefficient of determination of R^2^ = 0.95 for *H*_*PAR*_ (Fig. 5a), 0.96 for *M*_*PAR*_ (Fig. 5b), and 0.98 for *L*_*PAR*_ (Fig. 5c). All R^2^ values were highly significant (*p* < 0.0001). These results indicate that the proposed modelling approach predicted the behavior of sap flow fairly well in relation to microclimatic variables of different radiation intensities (i.e., agroforestry arrangements), with the coefficient of determination being equal or greater than 0.95 for all AFs (Fig. 5). In all AFs (or radiation intensities), the regression line was a bit below the line y = x for high *V*_*s*_ values and a bit above it for low *V*_*s*_ values (Fig. 5). When analyzing the relationship between observed and fitted values for the model, the RMSE ranged from 0.01 to 0.02 L h^−1^ for the different radiation intensities or AFs (Fig. 5). Likewise, the slope of the regression of the predicted vs. observed values in the different radiation intensities was statistically different from the reference slope (y = x) for all AFs (Fig. 5). This means that, in general terms, the model overestimates the values of *V*_*s*_ by an average of 2.8%.

## Discussion

This study presents a model that empirically determines the relationship between different microclimatic variables (*PAR*, *T*_*a*_, *RH*_*a*_, and *VPD*) and diurnal sap flow patterns in cacao plants under different agroforestry arrangements in the continental Amazon region. These arrangements mainly differed in their intensity of incident radiation on the cacao crop. Incident radiation had a direct impact on microclimatic conditions that greatly differed between AFs. There are few models that include a set of microclimatic variables to predict diurnal patterns of cacao sap flow. Studies such as those developed by Köhler et al.^17^ in Indonesia have also found that different microclimatic variables have significant effects on *V*_*s*_ in cacao plantations under different levels of sun radiation. The Köhler et al.^17^ modeling results were derived from a Jarvis-type sap flow model that found differences in sap flow response to changes in both vapor pressure deficit and radiation. The same occurred in López et al*.*^51^ in Mexico, which found that variations in temperature and sun radiation significantly affected transpiration rates (and thus, sap flow rates) when there were variations in cloud cover. Studies conducted by Abdulai et al*.*^52^ in Ghana showed the diurnal water use of the cacao crop as well as the average diurnal sap flow density, but did not specifically describe the use of models derived from environmental variables to predict sap flow. Overall, by predicting the diurnal pattern of cacao sap flow, the results from this type of modeling approach can also be used to obtain the diurnal patterns of transpiration and canopy conductance of cacao plants using the inverted Penman–Monteith equation as reported in different studies^14–16^.

Our model determined the relation between different microclimatic conditions and cacao sap flow during the relative dry season in the Amazonian region, a period of great agronomic importance for fruit set and cacao pod production. The prediction of sap flow and water relations of cacao crops during this relatively dry season is of great economic importance. The production of cacao beans depends largely on the amount and quality of the flowers during the blooming season^53^, which in the continental Amazonian region occurs at the end of this relatively dry, hot period^35,54,55^. Cacao plants tend to be particularly active during this season and display high photosynthetic rates in order to bloom appropriately. However, unlike other cacao producing tropical regions with high diurnal insolation, the average sunshine duration in the continental Amazon is only 3–4 h per day^56^, due to its high cloud cover. This situation causes different shade configurations in cacao agroforestry systems^57^ and, in turn, variations in microclimatic conditions^58^. These variations greatly affect cacao production^15^, particularly under the more shaded AF arrangements.

Our results showed that increases in *PAR* and *VPD* were positively correlated with sap flow values, while this correlation was negative between *V*_*s*_ and *RH*_*a*_. This indicates that microclimatic variables decisively influenced the quantity and direction of *V*_*s*_ during both day and night. The effect of the microclimatic conditions was modified by the upper tree canopy and plant density, which determined variations and differences in the pattern of *V*_*s*_ in each AF. The model accurately predicted *V*_*s*_ patterns at times during the day when sap flow was positive (i.e., flowed from the root system to the main trunk and branches). This prediction was particularly accurate for *H*_*PAR*_ during the entire monitoring period, where *V*_*s*_ increased from 0.039 L h^−1^ in the cacao tree trunk from 8:00 h until noon, reached its maximum level at 13:00 h (0.228 L h^−1^), and decreased to 0.031 L h^−1^ in the afternoon. This behavior was similar to that reported by Köhler et al.^17^ and López et al*.*^51^ for cacao trees in AFs under tropical wet climates in Indonesia and Mexico, respectively. The positive and more voluminous sap flow in *H*_*PAR*_ as compared to the other AFs suggests that air conditions (and probably soil humidity) were optimal at the high levels of radiation present in *H*_*PAR*_, which was directly related to the high demand for water of cacao trees so as to be physiologically active and perform photosynthesis^36^. In fact, the maximum photosynthetic rates in cacao trees in *H*_*PAR*_ were significantly higher than those in cacao trees in the *M*_*PAR*_ and *L*_*PAR*_ AFs^57^.

Besides predicting the positive sap flow in cacao plants and its differences between agroforestry arrangements, our model was also able to predict inverse or basipetal flows during the night. This inverse flow (from the main trunk to the roots and potentially to the soil) was measured in the agroforestry arrangements *L*_*PAR*_ and *M*_*PAR*,_ which exhibited values of − 0.04 and − 0.01 L h^−1^ during the night, respectively. Basipetal sap flow has been extensively described by Burgess et al*.*^59^ and many others, who consider this phenomenon to be directly related to plant hydraulic redistribution. Hydraulic redistribution is considered indicative of a passive process of sap movement in the direction of the maximum difference in water potential between the parts of the plant (branches, trunk, roots) and the soil^59,60^. In the *L*_*PAR*_ and *M*_*PAR*_ arrangements, it is possible that the cacao plants had a higher water potential (closer to zero) during the night than the surface soil layers, which may have dried during the day due to water uptake by the whole plant community of the AF and soil water evaporation. Soils in these shaded AFs might have been drier than in *H*_*PAR*_ (where there was more incident radiation, a higher temperature, and potentially more evaporation in the soil than in *L*_*PAR*_ and *M*_*PAR*_), probably due to the great demand for water by the companion plant species that make up the shade canopy in *L*_*PAR*_ and *M*_*PAR*_. Our results showed that the higher the density of companion plants (*L*_*PAR*_ > *M*_*PAR*_ > *H*_*PAR*_), the greater the hydraulic redistribution observed, suggesting there was a depletion in surface soil water content with increasing plant density. In fact, results from other studies support this idea, as soil humidity data measured in the same AFs during the dry season showed that surface soil profiles in the *L*_*PAR*_ and *M*_*PAR*_ AFs were, on average, drier than *H*_*PAR*_^61^. Moreover, the fact that our sap flow predictive models differed across AFs suggests that soil water content not only differed among AFs but also modulated cacao sap flow dynamics.

For this plant hydraulic distribution to occur, it is necessary that nocturnal transpiration stops or vastly decreases; in such cases, the highest water potential gradient in the plant soil continuum may be between the wet soil profiles and drier surface soil layers^62^ and, potentially, from the trunk to the roots^63^. Low *VPD* values (e.g., fog and cloudy nights with a high *RH*_*a*_) may impair nocturnal transpiration^62^, creating favorable conditions for water to be redistributed into dry soils. Our results align with this idea: in *L*_*PAR*_ and *M*_*PAR*_, unlike in *H*_*PAR*_, there were low levels of *VPD* and very high levels of *RH*_*a*_, which likely inhibited the nocturnal transpiration of cacao plants, allowing the preferential sap flow to occur from the plant to the relatively drier surface soil profile. The diurnal proportion of reversed *V*_*s*_ (i.e., the proportion of flow that went from the plant tissue to the roots and, potentially, on to the soil) was 49.3% in *L*_*PAR*_ and 5.08% in *M*_*PAR*_; these values fall within the range reported by other authors^63,64^ for other forest species. This ability of cacao to redistribute water is probably relevant to reduced competition for surface water with the companion species the next day, allowing cacao trees from *L*_*PAR*_ and *M*_*PAR*_ to be physiologically active during the next day. Instead, and like *L*_*PAR*_ and *M*_*PAR*_, the positive nocturnal sap flow exhibited by cacao trees in *H*_*PAR*_ is probably indicative of significant plant nocturnal transpiration, which usually inhibits hydraulic redistribution^60,65^. Significant nocturnal transpiration indicates that the maximum gradient of water potential for cacao plants in *H*_*PAR*_ would have been between the soil profile to the roots, and from the roots to the leaves and atmosphere, preventing (or reducing) hydraulic redistribution^66^.

## Conclusions

The different cacao agroforestry arrangements, which differed in their structure and density of shade canopy plants, generated different incident radiation intensities, modifying the microclimatic conditions in each plantation and directly affecting diurnal patterns of sap flow in cacao trees. We accurately modelled sap flow as a function of a set of microclimatic variables, including PAR, VPD, RH_a_, and temperature in each AF, and accurately modelled the differences in sap flow across AFs. Nonetheless, and regardless of the type of agroforestry arrangement, the variable with the greatest effect on cacao *V*_*s*_ was *PAR* (and thus, incident radiation), which also modulated RH_a_ and T_a_ values, having all an effect on *VPD*. The model can also track plant phenomena such as nocturnal transpiration (in *H*_*PAR*_) and inverse nocturnal sap flow indicative of hydraulic redistribution (in *M*_*PAR*_ and *L*_*PAR*_) and, thus, could be a useful tool for managing and predicting cacao tree water use as a function of the microclimatic conditions in the different agroforestry arrangements in the Continental Amazon rainforest region.
